# Current Perspectives on the Neurobiology of Drug Addiction: A Focus on Genetics and Factors Regulating Gene Expression

**DOI:** 10.5402/2012/972607

**Published:** 2012-10-14

**Authors:** Jhodie R. Duncan

**Affiliations:** ^1^The Florey Institute of Neuroscience and Mental Health, The University of Melbourne, Parkville, VIC 3010, Australia; ^2^Department of Anatomy and Neuroscience, The University of Melbourne, Parkville, VIC 3010, Australia

## Abstract

Drug addiction is a chronic, relapsing disorder defined by cyclic patterns of compulsive drug seeking and taking interspersed with episodes of abstinence. While genetic variability may increase the risk of addictive behaviours in an individual, exposure to a drug results in neuroadaptations in interconnected brain circuits which, in susceptible individuals, are believed to underlie the transition to, and maintenance of, an addicted state. These adaptations can occur at the cellular, molecular, or (epi)genetic level and are associated with synaptic plasticity and altered gene expression, the latter being mediated via both factors affecting translation (epigenetics) and transcription (non coding microRNAs) of the DNA or RNA itself. New advances using techniques such as optogenetics have the potential to increase our understanding of the microcircuitry mediating addictive behaviours. However, the processes leading to addiction are complex and multifactorial and thus we face a major contemporary challenge to elucidate the factors implicated in the development and maintenance of an addicted state.

## 1. Introduction

### 1.1. Impact of Drug Addiction on Society

Drug addiction is a serious socioeconomic problem associated with significant mortality and morbidity. Recent estimates suggest that in 2009 as many as 271 million people participated in drug use at least once worldwide, reflecting nearly 1 in 20 people aged between 15–64 years of age [1]. However these numbers do not include the use of 3,4-methylenedioxy-N-methylamphetamine (MDMA or ecstasy), hallucinogens, and inhalants suggesting this figure could be substantially higher. Results from the 26,000 people aged 12 years or older who participated in the 2010 National Drug Strategy Household Survey conducted by the Australian government indicated that 15.1% of people smoked daily, 7.2% consumed alcohol with 20–28% of these drinking at levels that would be considered harmful, 10.3% used cannabis, 4.2% used pharmaceuticals for nonmedical purposes, 2.1% used cocaine, and 1.4% used hallucinogens [2]. Drug use of these proportions places a great burden on society. Within Australia smoking equates for 7.8% of the burden of disease, alcohol consumption up to 3.8% and 2% for illicit drug use [3] with an estimate of over $56 billion in economic drug associated costs (approximately 56% for tobacco, 27% for alcohol, and 15% for illicit drugs) in 2004 to 2005 alone [4]. Of this, nearly $31 billion relates to tangible costs (expenses, wages) and $25 billion to intangible costs (diminished quality of life). Together this more than triples the costs of cancer and cardiovascular disease combined [5, 6]. Furthermore, it is estimated that drug-related issues consume up to 3.5% of the gross domestic product in western countries [7], which is equivalent to $485 billion in the United States of America alone [8]. There is also an association between drug use and psychotic episodes, violence and aggression, mood disorders, depression, paranoia, and suicide, which adds significantly to society's burden. What is more these figures also fail to account for the impact upon the user's family and friends.

### 1.2. Definition of Drug Addiction

Drug addiction, relating to both licit and illicit substances, is a chronic, relapsing disorder defined as compulsive drug seeking and taking that continues despite significant negative consequences [9]. Addictive behaviours usually begin with a period of experimentation with a particular drug, the use of which escalates over repeated exposures associated with the appearance of tolerance; that is, an individuals' hedonic (pleasure) set point increases and increasing amounts of the drug are needed to reach the “high” produced by earlier lower quantities (see [10]). As an individual heads towards dependence, there is an increase in the motivation to obtain and continue using a drug and a loss of control in limiting drug intake. There is also a growing awareness of the emotional consequences associated with drug use and a link to environments associated with accessing or taking the drug [11]. Following chronic drug use, many individuals go into withdrawal, usually through self-imposed abstinence, which may see the development of withdrawal syndromes in some patients [12]. Withdrawal is also often associated with a negative emotional state with individuals displaying high levels of depression, stress, and anxiety. In the majority of individuals (up to 90%) this is followed by relapse [13] resulting in a repetitive, cyclic drug taking pattern which displays a high degree of resistance to cessation of drug seeking (see [14]). Consequently, the progression from casual drug use to an addicted state is commonly referred to as the *cycle of addiction* which consists of 4 main stages: (1) preoccupation and anticipation of the drug, (2) intoxication, usually in a binge-like fashion with loss of control, (3) withdrawal, usually associated with negative affect, and (4) craving generally followed by relapse. 

However, not all individuals who experiment with drugs of abuse go on to become addicted, indeed it is estimated that only 15–20% of individuals who engage in drug taking will meet the criteria for dependence (see [1]). Furthermore, an individual's vulnerability to displaying addictive behaviours is highly complex and multifactorial being influenced by (epi)genetic, biological, and environmental factors [15, 16]. As a result, individuals who go on to display characteristic addictive behaviours may be influenced by factors mediating their vulnerability to initiate and engage in drug use which is further influenced by factors that mediate their shift from casual to compulsive drug use [15]. Addiction also involves components of both compulsivity and impulsivity; for example, premature responding, as a measure of high impulsivity on attentional tasks, is associated with cocaine seeking and relapse [17]. While drug use is associated with high impulsivity in humans [18] and may further exacerbate the impulsive nature of an individual [19], evidence from human studies suggests that trait impulsivity may play a greater role in the influence over an individual's predisposition to continue drug-seeking and -taking behaviours [20]. This includes resistance to ceasing these behaviours during abstinence as drugs known to reduce impulsivity may aid in the prevention of relapse [21]. Assessment of impulsivity in children and adolescents has been used to predict subsequent alcohol-related issues and drug use [22] and smoking vulnerability [23], with analysis of high impulsivity in siblings of cocaine users suggesting a heritable basis [20]. Impulsivity is also thought to precede compulsivity and it is the shift from impulsive to compulsive behaviours that is linked to the development of addiction in many individuals (see [24]). Impulsivity can also be distinct from other behaviours such as anxiety, response to a novel environment, and some forms of stress response [25]. However trait impulsivity appears to play greater role in the loss of control over some drugs such as cocaine compared to heroin, for example [26, 27]. 

Due to the complex nature of drug addiction, our greatest understanding of the neurobiological aspects of processes mediating addictive behaviours have, to date, largely come from preclinical studies in animal models. Both humans and animals will voluntarily consume drugs of abuse and display preferences for environments associated with exposure to a drug, continued motivation to obtain a drug, an inability to limit or stop taking a drug despite adverse consequences, and a high incidence of relapse following withdrawal [28]. Animal models can also be assessed based on genetic variability, which indicates an association of heritable factors such as impulsivity, with the predisposition to engage in drug-seeking behaviours as seen in the human situation [24]. Of relevance, like humans, only a subset of animals exposed to a drug (~17%) will display addictive-like behaviours [28] providing validity in the extrapolation of animal data to the human situation.

This paper will provide an overview of the current knowledge base of the complex mechanisms mediating addictive behaviours, incorporating the literature from both human studies and preclinical animal models. Rather than in-depth exploration of any one topic, it will highlight the key processes that are believed to drive continued drug use by presenting specific examples of the neuroadaptive changes that exist following acute exposure to a drug and how these may differ following repeated exposures and following periods of withdrawal. The paper will also explore the molecular and cellular mechanisms linked to substance abuse with a particular focus on genetics, epigenetics and noncoding RNAs, and the recent advances in our understating of their role in addiction. Finally, the paper will introduce the concept of optogenetics and highlight how this technique may be of value to addiction neuroscience. 

### 1.3. Circuits and Neurotransmitters

There are many different drugs that are commonly abused in society, some being obtained legally such as alcohol, tobacco, and inhalants while others are obtained illegally, such as marijuana, amphetamine, cocaine, and heroin. Each drug has a differing chemical and pharmacological profile, can be administered via different routes, acts on different neurobiological systems, and undergoes different metabolic pathways [29]. Nevertheless, all drugs appear to exert their initial effects on the mesocorticolimbic dopaminergic pathway which is heavily involved in reinforcement learning [30]. Originating from dopaminergic neurons located in the ventral tegmental area (VTA) of the midbrain this pathway projects to the nucleus accumbens (NAcc) (motivation), amygdala (mediates association of reward with cues and negative reinforcement), ventral pallidum, hippocampus (limbic system associated with memory and learning), and forebrain, in particular the prefrontal cortex (PFC). The acute exposure to a drug results in a transient increase in the extracellular levels of dopamine in the NAcc [31] and other projection sites, though the process via which this occurs may be dependent on the drug itself. For example, for alcohol, nicotine, opiates, cannabis, and inhalants this occurs via enhancement of dopamine release from presynaptic terminals primarily as a consequence of increased neuronal firing in the VTA [32]. In comparison, for cocaine this occurs via the inhibition of presynaptic dopamine uptake by the dopamine transporter (DAT) or via actions on the vesicular monoamine transporter which actively reverses DAT function to elevate extracellular dopamine in the NAcc, which is the case for amphetamine [33]. Following repeated exposure to a drug there is a progressive increase in basal levels of dopamine and the appearance of tolerance [34] along with altered dopamine receptor expression [35]. Should exposure to the drug cease, the levels of dopamine typically fall below normal baseline levels. Conditioned cues associated with drug taking can be sufficient to induce relapse and indeed these cues are powerful enough to activate dopaminergic brain regions in patients who regularly abuse cocaine [36].

However, the VTA contains a heterogeneous population of cells with approximately 65% being dopaminergic, 30% being gamma-aminobutyric acid (GABA)ergic, and 5% being glutamatergic (see [37]). The primary afferents to the VTA are excitatory glutamatergic inputs from the PFC and inhibitory GABAergic inputs from medium spiny neurons (MSNs) in the NAcc, both of which can form feedback loops to regulate activity in this region though this can occur via different mechanisms. Drug-induced release of GABA, for example, may mediate local inhibitory feedback mechanisms by binding presynaptically to receptors expressed by MSNs themselves or enhance synaptic inhibition by binding postsynaptically at projection sites [38], thus causing disinhibition of downstream targets. This includes increasing dopamine release, as the firing rate of VTA neurons is under inhibitory control [39]. The VTA also receives connections from regions such as the ventral pallidum, hippocampus, amygdala, lateral hypothalamus, and bed nucleus of the stria terminalis [40, 41]. 

Aside from the VTA, the NAcc is believed to play a substantial role in mediating addictive behaviours [42, 43]. The NAcc is involved in the control of the motivational value of stimuli [44], reward reinforcement [45], and the mediation of impulsive choice [46]. As well as dopaminergic inputs from the VTA, the NAcc also receives excitatory glutamatergic input from the PFC, amygdala, thalamus, and hippocampus thus serving as an interface between the limbic (processing of new and learnt information) and motor (task performance) pathways. Furthermore MSNs, the dominate cell type in the NAcc, can express either dopamine D_1_ receptors and thus form part of the direct pathway that results in neuronal excitation or D_2_ receptors and thus form part of the indirect pathway that results in neuronal inhibition [47, 48]. Consequently, the activation of these receptor subtypes and thus modulation of the direct and indirect pathways determine which signals are reinforced and which are suppressed [49]. More recently human functional neuroimaging studies have identified that the PFC plays an important role in addiction [50]. This is thought to occur, in part, via the PFCs ability to regulate the activity of neurons in reward associated nuclei (i.e., the NAcc and VTA) and its involvement in inhibitory control over behaviour, planning, and executing complex cognitive behaviours and executive function (i.e., awareness, decision making, and self-control) [50]. The PFC is also believed to play a pivotal role in mediating controlled drug intake with a transition from PFC to striatal control as drug use transfers into a compulsive behaviour (see [51]). However, the highly integrated networks mediated by exposure to a drug of abuse and the different neurotransmitter systems they may act upon permit considerable crosstalk which acts to fine tune signal transmission, with the resultant outcome being driven by an integration of all incoming signals.

## 2. Neuroadaptations and Addiction

### 2.1. Plasticity

There are many ways to investigate drug-related processes in animal models, one of the most common being via a self-administration paradigm. This paradigm requires an animal to perform a task, such as pressing a lever, which results in access to a drug. This motor task is normally associated with a conditioned stimulus, such as a light cue, that becomes associated with the drug reward. The conditioned stimulus is used to mimic the effects of environmental associations with drug use that exist in drug addicted humans. This model can be used to assess different components of addictive behaviours including spontaneous initiation of drug taking, motivation, persistence and relapse, and the neural pathways mediating these responses (see [52]). Using self-administration paradigms, studies like those by Porrino et al. have reported that the functional activity in the brain of rhesus monkeys self-administering cocaine is altered when compared to those receiving a food reward. Initially activity is noted in regions mediating motivation and reward, such as the ventromedial PFC and ventral striatum, which extends to regions involved in emotion and cognitive processing upon extended repeated exposures (100 sessions) [53]. Of interest, in this study the changes in functional activity induced by exposure to cocaine were still present after one month of abstinence suggesting that drug-induced plasticity and long-term adaptations had occurred. These data support reports from human studies of patients with drug addictions where the structures contributing to the corticostriatal system are abnormal on magnetic resonance imaging (MRI) scans which is subsequently associated with altered behavioural performance in these individuals [54].

As not all individuals who engage in drug use become addicted, it is believed that, in the subset of individuals who do become addicted, exposure to a drug is sufficient to result in neuroadaptive changes to drug sensitive pathways in the brain that extend beyond the initial neuropharmacological actions of the drug. Following subsequent exposures these neuroadaptations, at either the cellular, synaptic, molecular or (epi)genetic level, lead to altered behaviour and the driving force to continue drug-seeking and -taking behaviours (see [41, 55]). Examples of the mechanisms mediating one of the commonest forms of drug-induced neuroadaptive change, that is, synaptic plasticity, are highlighted below.

### 2.2. Synaptic Plasticity

Drugs of abuse are able to induce either pre- or postsynaptic plasticity, a process which is mediated in an activity-dependent manner. This can occur via changes to synaptic structures themselves, via effects on signal transduction, or at the level of receptor expression, all of which have the potential to lead to synaptic rearrangement and altered neuronal signaling. 

#### 2.2.1. Dendritic Spines

Changes to the density and morphology of dendritic spines is a commonly reported neuroadaptation following exposure to a drug of abuse or during withdrawal itself. Golgi-Cox staining has illustrated a significant increase in the density and a 2.6-fold increase in branching of dendritic spines of MSNs in the shell of the NAcc following abstinence from cocaine for one month after one month of self-administration in rats [56]. A similar change is observed at pyramidal cells in the PFC and parietal cortex. Changes to dendritic spines may be specific in nature. Kim et al. have shown that cocaine repetitively injected into transgenic mice expressing green fluorescent protein under the control of promoters for either D_1_ or D_2_ receptors results in a selective increase in spine density for MSNs expressing D_1_ but not D_2_ [57]. This correlates with decreased membrane excitability in these neurons, with no corresponding change observed in D_2_ expressing MSNs. This highlights that neurons within the same cell population may differentially contribute to a drug-induced neuroadaptation. Drug-induced changes to dendritic spines may also occur via separate but interactive signaling pathways resulting in more than one structural alteration [58] having the potential to cause regional, compartmental, or type-specific changes to dendritic spines themselves [59].

#### 2.2.2. Neurotrophic Factors

The neurotrophin brain-derived neurotrophic factor (BDNF) and its interactions with TrkB receptors (its primary receptor) are believed to play an important role in drug-induced neuroadaptive changes and therefore addictive behaviours [60–62]. Cocaine-dependent individuals have high serum levels of BDNF which have been found to be predictive for increased risk of relapse following a period of abstinence up to 90 days [63], with elevated levels being maintained up to 6 months into abstinence in prior alcohol-dependent patients [64]. Furthermore, in animal models, there is a progressive increase in the levels of BDNF in reward-associated nuclei (VTA, NAcc, and amygdala) during 60 days withdrawal from cocaine, which is associated with incubation of craving (see below) in these animals [65]. Levels of BDNF in the NAcc have also been linked to enhancement of locomotor activity, conditioned place preference (CPP), and reinstatement following cocaine [66].

Drug-induced changes to BDNF levels in mice occur in a time-dependent manner, occurring in the NAcc prior to the VTA [60]. The authors suggest that time-dependent increases in BDNF may lead to synaptic modifications that enhance cue-induced reinstatement following extended periods of withdrawal and implicate BDNF in persistent drug-seeking behaviours, at least for cocaine [67]. Both the levels of BDNF, the density of dendritic spines are altered following exposure to ethanol in rats [68] and it is the changes to BDNF that are, in part, believed to mediate changes to dendritic spines as BDNF can be locally synthesized within the spine itself [69]. *In vitro *experiments in cultured rat hippocampal neurons have shown that exposure to BDNF activates TrkB leading to downstream signaling cascades and the promotion of neurite elongation, and enlargement of the head of dendritic spines [70]. Should TrkB activation be sustained subsequent neurite branching and neck elongation are observed. 

#### 2.2.3. Signal Transmission

 BDNF/TrkB interactions also enhance basal levels of long-term potentiation (LTP) in cultured hippocampal neurons [70]. This suggests that BDNF may also play an important role in modulating activity-dependent signal transmission between neurons [71]. There are two markers of long-term activity-dependent signal transmission, either LTP or long-term depression (LTD). LTP refers to an enhancement of signals between neurons and is associated with an increase in synaptic strength. In contrast, LTD refers to a suppression of signals between neurons and is associated with the weakening and elimination of synapses. Consequently, it is the combined actions of LTP and LTD that refine and consolidate adaptive changes to neuronal circuits [72, 73]. Both LTP, and more recently LTD are emerging as key players in the mediation of addictive behaviours. 

#### 2.2.4. LTP

A single acute exposure to cocaine in rat slice preparations is sufficient to induce LTP at glutamatergic synapses in the VTA [74] and repeated inductions of LTP leads to synaptic enhancement [75]. After 3 inductions of LTP, via exposure to glutamate each 24 hours apart, there are long-lasting and time-dependent changes to the expression of synapse-related genes occurring at either 24–96 hours or 6–12 days after the last exposure [76]. Interestingly, in dopaminergic neurons, exposure to cocaine inverts the processes that lead to the generation of LTP from depolarization to hyperpolarization [77]. Self-administration studies have shown that drug-induced potentiation, the effects of which were greater for cocaine over natural food rewards, persists during abstinence for up to 3 months [78]. The same effects are not observed if cocaine is passively infused suggesting that the drug itself is insufficient to induce this change and that associated cues play a role in these adaptations. Drugs also appear to alter the ability to subsequently modulate signal transmission as during withdrawal from cocaine there is an inability to develop further LTP and LTD in the NAcc after stimulation of the PFC [79]. The authors show that this effect can be restored by application of N-acetylcysteine, which increases glutathione synthesis thus activating the cystine-glutamate exchanger and restoring glutamate homeostasis. 

#### 2.2.5. LTD

There is also growing evidence that long-term impairments in LTD may play a role in the maintenance of an addicted state. In animals that are sensitized to cocaine there is long-lasting depression at excitatory synapses made by PFC afferents onto MSNs in the NAcc shell [80]. However LTD does not appear to be impaired during the initial phase of learning in a cocaine self-administration paradigm, but once learning has been consolidated, LTD is suppressed [81] and remains suppressed in the NAcc even during the initial stages of abstinence [82]. In animals that do not display the hallmarks of addiction deficits in LTD progressively recover even though these animals maintain a controlled drug intake. However in animals that do display behavioural hallmarks of addiction LTD remains suppressed [81]. The same group has gone on to show that this effect can be related to LTD mediated by stimulation of specific receptor subtypes (endocannabinoid mediated versus glutamatergic) in the PFC [83]. In rats, blocking LTD at glutamatergic synapses in the NAcc is sufficient to prevent the expression of amphetamine-induced behavioural sensitization [84]. A single presentation of both the *N*-methyl-D-aspartic acid (NMDA) receptor antagonist 2-amino-5-phosphonovalerate and the group I/II metabotropic glutamate receptor (mGluR) agonist 1-aminocyclopentane-1,3-dicarboxylic acid is sufficient to stimulate a transient LTD, which normalises within 24 hours [85]. However, repeated inductions of LTD lead to a persistent decrease in excitatory postsynaptic potentials and the number of synaptic structures. In support of these observations, Egashira et al. have shown that 3 inductions of LTD lead to reduced synaptic strength and the number of pre- and postsynaptic structures. This process requires rapid novel protein synthesis (within 6 hours) [86] the effects of which can last up to 14 days after the last induction [87]. Consequently LTD is also believed to play a role in relapse, which may be dependent on the nature of how the drug is removed. Both straight withdrawal and extinction training (see below) following cocaine self-administration result in reduced LTP in the NAcc following stimulation of the PFC, though only extinguished animals have blunted LTD [88].

#### 2.2.6. Receptor Expression

Drugs of abuse may either increase or decrease the expression of receptors on cells or their processes [89], with subsequent changes occurring during periods of abstinence [90], in a dose-dependent and region-specific manner [90]. Cocaine-related fatalities have been associated with an increase in the mRNA for dopamine D_3_ receptors in the NAcc [91] while in drug abusers with a history of cocaine use, heroin use, or both, there is a strong positive correlation between the expression of mRNA for the A1 subunit of amino-3-hydroxy-5-methylisoxazole-4-propionic acid (AMPA) glutamate receptors and scaffolding proteins such as the post-synaptic density protein-95 in the amygdala [92]. The authors argue that this response is indicative of strengthening of synapses in this region. In animal models, acute exposure to cocaine can transiently and rapidly reduce the expression of mGluRs in the striatum with levels returning to normal by 6 hours after exposure and no associated change in the PFC or hippocampus [93]. In the VTA, AMPA GluA1 subunits are decreased following acute exposure to cocaine in mice but only in the parabrachial region [94], in the paranigral region of the VTA the surface expression of these receptors is increased. Following 14 days exposure to cocaine, these responses are diminished in both regions but only for dopaminergic processes, in comparison GluA1 subunits on GABAergic processes display region-specific changes [94]. In both rats and mice after 3 weeks of withdrawal following repeated cocaine administration for 7 days there is an increase in both NMDA GluN2B and mGluR_1a_ receptors in the PFC, while different subunits of these receptors (GluN2A and mGlu_1_) are increased in the hippocampus [90]. The NMDA GluN2A subunit is also increased in the dorsal striatum but mGluR levels are not altered, and all 3 subunits (NMDA GluN2A, 2B, and mGlu_1_) are reduced in the shell of the NAcc. 

The ability to understand the complex nature of receptor-mediated adaptive changes following exposure to a drug is further heightened by the fact that receptors may be activated by more than one mechanism and/or ligand and may form oligomeric complexes with other receptors in a region-specific manner [95]. For example, some subtypes of mGluRs (i.e., mGlu_1_, mGlu_3_, and mGlu_5_) are responsive to not only glutamate (a neurotransmitter) but also extracellular levels of calcium (a divalent cation) [96]. This occurs via partially overlapping binding sites, with increasing calcium levels resulting in a greater prolongation of glutamatergic responses [97]. In cells mutation of the binding site for glutamate leaves calcium signalling intake; however, inhibiting calcium binding via similar methods reduces the sensitivity to both calcium and glutamate [98] indicating that functional calcium signalling is required for efficient glutamate signalling in some cases. It is hypothesised that these receptors work synergistically to yield a maximal effect so that the activities of downstream messenger systems mediating functions such as LTP are maintained. 

Heterooligomeric complexes have been shown to exist between dopamine D_2_ and mGlu_5_ receptors [99], mGlu_5_ and NDMA receptors [100], serotonin (5-HT)_2A_ and mGlu_2_ receptors [101], and mGlu_5_ and GABA(A)*α*1 receptors [102] as well as other combinations including adenosine A_2A_ [103], *μ*-opioid [104], or estrogen [105]. Recent evidence by Cabello et al. also suggests that higher order oligomers also exist. Using biomolecular fluorescence complementation, bioluminescence resonance energy transfer and sequential resonance transfer techniques complexes containing dopamine D_2_, mGlu_5_, and the adenosine A_2A_ receptors have been identified [106]. All 3 receptors are physically linked in the cell membrane and are expressed on post-synaptic dendritic spines of GABAergic striatopallidal neurons [106]. 

The presence of heteromeric complexes and higher-order oligomers aids in the explanation of the ability of a single neurotransmitter to activate and independently regulate several different pathways even though the receptors may be from different families [107]. This leads way to the possibility of synergistic and antagonistic interactions of different ligands at the one site to facilitate neurotransmitter release and induce synaptic plasticity in response to a drug of abuse. For example, perfusion of the mGlu_5_ agonist (RS)-2-chloro-5-hydroxyphenylglycine (CHPG) into the NAcc increases GABA release in the ventral pallidum [108]. This response can be potentiated by administration of CGS 21680, an adenosine A_2A_ agonist, and counteracted by coinfusion of quinpirole, a dopamine D_2_ agonist. These receptor complexes may also work synergistically to produce their resultant effects. Coadministration of subthreshold doses of the mGlu_5_ allosteric antagonist 3-((2-methyl-4-thiazolyl)ethynyl)pyridine (MTEP) and the adenosine A_2A_ antagonist SCH58261 is sufficient to reduce ethanol responding and cue-induced reinstatement of ethanol seeking [109]. The same effects are not observed when administered alone or if these drugs are combined with 8-cyclopentyl-1,3-dipropylxan (DPCPX), an adenosine A_1_ antagonist suggesting subtype receptor specificity of these interactions. Furthermore, costimulation of the adenosine A_2A_ and mGlu_5_ receptors results in a synergistic effect on extracellular signal-regulated kinase 1/2 (ERK) phosphorylation and cFos expression [103]. While the authors did not observe a synergistic effect at the level of second messenger systems in this study, others have used mice neostriatal slices to show that synergistic responses do occur at this level which potentiates the formation of 3′-5′-cyclic adenosine monophosphate (cAMP) and increases phosphorylation of the signal transduction molecule dopamine- and cAMP-regulated phosphoprotein of 32,000 kDa (DARPP-32) involved in dopaminergic signalling [110].

### 2.3. Impact of Drug Induced Neuroadaptations

As a result of the above-mentioned processes, drug-induced neuroadaptations alter homeostatic set points and thus the ability of the brain to function “normally”, reducing the ability to process the adverse consequences of drug taking. Neuroadaptations also play a role in the lack of flexibility in the control over addictive behaviours which results in a window of opportunity before drug-related behaviours switch from goal-directed actions that are still sensitive to devaluation to habitual-based responses [51, 111]. This corresponds to a neuroadaptive shift in the brain regions mediating these responses. Consequently neuroadaptations are believed to underlie the switch from voluntary controlled drug use to habitual, compulsive drug use and the maintenance of drug-seeking behaviours. They appear stable and persistent and are linked to the high incidence of relapse following a period of abstinence (for reviews see [72, 73]). Drug-induced neuroadaptations are also believed to lead to “incubation of craving”, a complex phenomenon where drug-seeking behaviours increase over time being associated with not only exposure to the drug but also drug-associated cues. This phenomenon has been shown in animal models were the responsiveness to drug-paired cues progressively increases [65, 112]. For example, in rats responding for cocaine in a self-administration paradigm is significantly increased by day 7, compared to the first day, of abstinence following only 10 days of prior exposure, which progressively increases over 60 days of abstinence [112] and is maintained for up to 100 days of abstinence [113]. 

Drug-associated cues can potently drive relapse during a period of abstinence [114]. Consequently it is hypothesized that abstinence alone is insufficient to reverse drug-induced adaptations suggesting a *persistent* alteration of neuronal plasticity. Indeed prior exposure to a drug may actually be sufficient to inhibit the ability of stimuli to induce subsequent neuroadaptive changes [79, 115]. In this regard, there is growing evidence in animal models of the effectiveness of extinction training over withdrawal paradigms in maintaining abstinence [116]. During extinction training drug-seeking actions do not yield a drug reward, removing the adaptive value of drug seeking (comparable to cue exposure therapy during rehabilitation in humans) (see [117]). As extinction training results in altered molecular mechanisms that are not observed following abstinence alone [88], it appears that old behaviours remain intact and are not simply “unlearned”, rather new pathways and subsequently contextual behaviours need to be acquired in order to maintain abstinence [118]. In both human and animal studies, the effectiveness of extinction training can be enhanced via the incorporation of active retrieval of drug-associated memories prior to a training session [119]. In this manner the brain needs to form new adaptations and learn new memories other than those associated with drug-seeking and -taking behaviours that consolidate the experience of “no drug”. However extinction training does not appear effective in altering the enhanced LTP observed following exposure to cocaine which persists in the VTA after 3 weeks of abstinence and following cue-induced reinstatement and, thus even when drug-seeking behaviours are extinguished, neurotransmitter function remains potentiated (at least for glutamatergic processes), potentially driving drug-seeking behaviours [78]. 

## 3. Genes and Addiction

### 3.1. Genetic Links to Dependence

Addiction is a complex disease that is multifactorial and polygenic, thus it does not follow a clear Mendelian pattern of gene expression. Unlike disorders such as Down's syndrome, no single gene has been identified that predisposes individuals to develop addictive behaviours. However, genetic background is believed to influence addiction liability with mutations in certain genes believed to increase an individual's vulnerability to addictive behaviours should they engage in drug use (see [120]). Twin and adoption studies indicate the heritability of genes that predispose an individual to becoming addicted ranges from 40% to 70% (alcohol: 50%, cocaine 60%, and opiates 70%) [121]. That is up to 70% of the risk for addictive behaviours can be attributed to heritable influences, with a 4–8-fold increase in the risk of developing an addictive behaviour if a first-degree relative has a substance abuse disorder (see [122]). To date 1,500 genes have been linked to an “addiction” phenotype in humans [123] which can further be classified as those related to the initial stages of experimentation, those related to neuroadaptations following continued exposure, and those that influence outcome including the age of onset and patterns of use. The influence of genetic background over addiction appears additive; the more mutations an individual has, the greater their vulnerability to becoming addicted with different genes contributing to addictive behaviours in different individuals is (see [124]). However environment may also exert considerable influence over the development of addiction, which is further confounded by gene-environment interactions [125]. Many psychiatric disorders including depression, stress, and anxiety coexist with substance abuse disorders, as such heritable factors that influence these disorders may be perpetuated by adverse environmental exposures which may act as a trigger to activate a genetic predisposition and influence behavioural outcome, that is, it increases the risk of substance use (see [122]).

The most common and best understood effects to the genetic sequence occur at the level of a change to a single nucleotide in a gene (SNP—single nucleotide polymorphism). There is growing evidence that SNPs have the potential to influence addictive behaviours, especially for alcoholism. SNPs in genes for the catalytic enzymes for alcohol metabolism including alcohol dehydrogenase 1B (which oxidises ethanol to acetaldehyde) or aldehyde dehydrogenase-2 (which converts acetaldehyde to acetate) are sufficient to reduce the risk for alcoholism in some populations [126]. Mutations in genes encoding the NMDA GluN2A subunit (Schumann et al., 2008), mGlu_5_ receptors [127], or GABA_A_ receptors [128] may lead to a higher risk of developing alcohol dependence. Individuals with variations in the genes for dopamine D_2_ receptors show younger onset and severity of drinking [129]. SNPs in genes encoding for the Y2 receptor for neuropeptide Y have been associated with alcohol dependence, alcohol withdrawal, comorbid alcohol use, and cocaine dependence [130]. In comparison, SNPs in genes for neuropeptide Y5 receptors are only associated with alcohol withdrawal in human studies [130]. See Dick and Foroud for an extensive review on polymorphism associated with alcoholism [124]. 

There is growing evidence that SNPs may also influence other addictive behaviours beyond those for alcoholism. A meta-analysis of the A1 allele frequency at the dopamine D_2_ receptor in drug addicted compared to control patients indicated an association with SNPs in this allele with alcohol, nicotine, and opiate dependence, but not stimulant use disorders [131]. Variants upstream of CHRNB4, which encodes for the beta 4 subunit of neuronal nicotinic receptors, have been linked to the age at onset for daily cigarette smoking [132]. SNPs in the *D1* gene (rs686) [133], *D2* gene, though weak (rs7131056, rs4274224, rs4648318, and rs6278) [134], and *D3 *gene (rs6280) [135] have also been associated with increased nicotine dependence. While other SNPs, including those in the *dynamin 1 *gene (rs3003609) involved in the control of synaptic endocytosis, have been associated with the extent of smoking (quantity and heaviness) [136]. Interestingly assessment of a similar SNP in the gene for the dopamine D_1_ receptor was not associated with methamphetamine abuse, though this and other studies highlight the need for ethnic considerations when comparing polymorphisms across racially diverse groups [137, 138]. SNPs in different opioid receptors and peptides have also been associated with different drug phenotypes (see [139] for extensive review). Val66Met polymorphisms for BDNF are associated with a current or former history of smoking [140] and the onset of heroin use [141], but not alcohol dependence [142]. In mice, this polymorphism has been shown to play a role in the extinction of cocaine responding but not reinstatement [143]. 

While 5-HT appears as an emerging factor in regulating addictive behaviours, associative studies in humans between SNPs in genes for either 5-HT_1B_ receptors [144] or the gene for tryptophan hydroxylase (the rate limiting enzyme for 5-HT synthesis) [145] have failed to be linked to cocaine or alcohol dependence. However, polymorphisms in the gene encoding the 5-HT transporter have been linked to sociopathy in alcoholics, supporting a role for 5-HT in mediating drug-related affective disorders [146]. Changes to the serotonergic system (either 5-HT levels or receptor expression) following exposure to a drug have been hypothesised to contribute to the high rate of affective disorders such as depression associated with drug abusers. It is these affective disorders which are thought to contribute to 5-HT's role in mediating drug-seeking behaviours with deficits in 5-HT transmission during the early stages of abstinence hypothesised to contribute to relapse in human patients [147]. 

Together these findings provide insight into genetic variability and its role in the manifestation of an addictive behaviour. Thus they provide novel targets and a potential short list of candidate genes to aid in refining treatment strategies. However the interpretation of these findings should not be over simplified. To date 31 SNPs for D_1_ receptors alone have been identified (see [131]) making direct linkages to aspects of addictive behaviours difficult. Indeed the relationship between SNPs and drug addiction is often weak and unlikely to explain all complex behaviours associated with the disorder. Add to this the possibility of structural variants (small rearrangements in the chromosome characterised by deletion or insertion of up to 1000 bp) and copy number variants (large rearrangements in the chromosome characterised by deletion, duplication, or insertion of over 1 Kb), both of which have not been assessed in relation to addiction, and the complexity increases several folds.

### 3.2. Gene Expression

Irrespective of the presence or absence of genes that may increase an individual's vulnerability to become addicted, human postmortem studies have shown that the expression of numerous genes is altered in the brain of addicted patients [148]. Chronic cocaine use, for example, differentially alters the expression of up to 49 transcripts in the NAcc including those involved in signal transduction, synaptic function, and DNA to RNA processing [149]. Similarly, significant upregulation of numerous transcripts, including glutamate receptors, have been reported using targeted microarrays on the VTA from cocaine overdose victims [150]. Importantly changes to transcripts reported following chronic cocaine use correspond to altered protein expression in both of these studies [149, 150]. In support of the observations in humans, up to 295 genes were differentially regulated after 1 h of nicotine treatment in cultured neuron-like SH-SY5Y cells, including those involved in neural development, synaptic plasticity, neuronal survival, immune responses, and cellular metabolism [151]. Indeed, genes affected by drugs of abuse can be subcharacterised into those relating to the extracellular matrix, synaptic plasticity and efficacy, receptors, ion channels and transporters, signal transduction, and cell death, among others (see [152] for an extensive list). 

While drugs of abuse may alter gene expression, consequently having impact on protein expression, the long-term effects are not fully understood. The mRNA for the immediate early genes cFos, Nr4a1, activity-regulated cytoskeleton-associated protein, and early growth response protein 1 are reduced in the PFC and NAcc during withdrawal from cocaine self-administration for 10 days in rats, the effects of which persist 100 days into withdrawal [113]. In contrast the mRNA for the neuropeptides cocaine and amphetamine-regulated transcript (CART) and neuropeptide Y were significantly increased following the first day of withdrawal only in the medial PFC. Chronic exposure to a drug may have a differential effect on the expression of these genes; indeed some genes show a sensitized response and others show desensitization. Furthermore, additional genes that had not previously been responsive to cocaine may show altered expression during abstinence [153]. Similar differential changes are observed during abstinence on protein expression [154]. Irrespectively these changes have the ability to affect behaviour, for example, there is a persistent downregulation of genes related to synaptic plasticity in the striatum of animals that are vulnerable to relapse to cocaine seeking [155].

Understanding the relationship of altered gene expression and its ability to mediate addictive behaviours is further complicated by the possibility of gene splicing. As proteins are not derived from continual sequences in the gene, splicing occurs when different exons of the RNA are reconstituted in different ways thus producing different isoforms of the same protein. For example, the genes encoding BDNF in rats can form 8 unique transcripts via slicing of four 5′ exons (exons I–IV) and one 3′ exon (exon V) [156], though up to 8 exons may exist [157]. Each exon may be differentially regulated to form distinct BDNF transcripts in response to local stimuli, including neuronal activation, in a developmental [158] and region-specific manner [159]. Furthermore, upon stimulus different splice variants of BDNF may be found in different subcellular compartments, including dendrites, in which they are not normally present under basal conditions [160]. In human postmortem studies, chronic cocaine use decreased the expression of BDNF4 and BDNF1 in the cortex, while it increased BDNF4 in the cerebellum relative to noncocaine users [161]. In the adult rodent brain all splice variants are expressed at high levels in the hippocampus, with splice variants for BDNF4 and 5 also being high in the striatum [157]. There is a rapid and dramatic (up to 4-fold) increase in the expression of the mRNA for BDNF4 in the striatum and PFC following acute exposure to cocaine; the splice variant for BDNF1 is also increased in cortex. However, the expression of all splice variants is unaltered following chronic exposure to cocaine for 10 days or following up to 3 months of abstinence [157]. This is in contrast to the observation that there is a progressive increase in BDNF levels in reward-associated nuclei (VTA, NAcc, and amygdala) over a prolonged 90-day period of withdrawal after cocaine self-administration [65], suggesting that additional factors may be acting to regulate the translation of mRNA to protein. There is also the possibility that SNPs in splice variants of a gene can influence addictive behaviours. The mRNA for dopamine D_2_ receptors exists as two splice isoforms, one of which is long (D_2_L) and one of which is short (D_2_S), being expressed primarily, but not exclusively, postsynaptically, and presynaptically, respectively. Human postmortem studies have shown that a history of cocaine is associated with 2 intronic SNPs (rs2283265 and rs1076560) in D_2_S which leads to a reduction in D_2_S and thus alters the D_2_S/D_2_L ratio in the PFC and putamen [138].

Patterns of gene expression are regulated by transcription factors which are transiently expressed to act locally to regulate gene expression at the cell in which they were initially produced. Transcription factors are proteins which bind to a specific DNA sequence to regulate the transcription of DNA to mRNA, often acting to enhance or retard the activity of enzymes such as RNA polymerase or molecules that regulate acetylation of histones (see below). Two common transcription factors associated with addiction are cAMP response element binding (CREB) and members of the Fos family. CREB is stimulated by initiation of the cAMP pathway in response to activation of receptors or ion channels. This leads to rapid yet transient CREB activation via phosphorylation (pCREB) which binds to a cAMP response element region in the DNA sequence and forms a complex with CREB-binding proteins to subsequently regulate gene transcription. CREB is expressed at high levels throughout the brain [162] and targets genes including cFos, BDNF, tyrosine hydroxylase, and several neuropeptides including corticotropin-releasing factor and dynorphin. 

Investigations into the role of CREB in mediating addictive behaviours have shown that overexpressing CREB in the NAcc alters drug-related behaviours including decreasing the rewarding effects of cocaine [163]. In contrast, increased expression of CREB in the NAcc shell enhances the reinforcement of cocaine self-administration in rats, independent of learning the task, and the motivation to obtain drug, the latter observation being correlated to modulation of BDNF protein levels in the same region [45]. The same group also showed in this study that increasing CREB in the NAcc following a period of abstinence enhances drug-primed reinstatement specifically but not cue- or stress-induced relapse. CREB in the dorsal striatum has also been linked to sensitivity to psychostimulants [164]. When mice are exposed to nicotine in their drinking water for 50 days the levels of pCREB are unaltered, however, after 24 hours of withdrawal there is a differential response to pCREB levels in reward associated nuclei [60]. In the NAcc the expression of pCREB is increased which corresponds to increased BDNF levels in this region, while in the VTA the levels are decreased relatively to those during chronic exposure to nicotine [60], the authors suggesting that this reflects compensatory responses in an attempt to maintain nicotine-induced adaptations in this pathway. The role of CREB in corticostriatal processes mediating specific elements of addictive behaviours should also be considered as conditional ablation of CREB1 in the cortex and hippocampus in mice decreases the motivation to self-administer cocaine, though animals still display a CPP and normal locomotor responses [165]. 

The Fos family of transcription factors include cFos, FosB, and ΔFosB (a splice variant of FosB) and represent an immediate early gene family which are rapidly induced following stimulation. They dimerise with proteins from the JUN family to form the AP-1 complex which consequently upregulates transcription. This complex can be activated via either extracellular signals or intracellular mechanisms such as phosphorylation. Fos transcription factors are believed to act as a molecular switch that leads to downstream consequences including altered protein expression, structural changes, and ultimately function (see [166]). Drugs of abuse have the ability to increase the expression of this family of transcription factors and while this occurs primarily in the NAcc, changes may also occur in the PFC, VTA, and amygdala, among others ([167], see [168]). Following chronic exposure to cocaine self-administration (18 days, for 4 hours a day) the expression of cFos, FosB, and ΔFosB is altered in a regional and temporal manner [169]. The expression of ΔFosB protein is the greatest in the NAcc; however, there is no difference between self-administrating and yoked animals indicating that this increase occurs in response to the drug itself and is not dependent on instrumental learning [169]. While responses can be sustained for extended periods [170], the expressions of FosB and cFos, however, show tolerance following repeated exposure to cocaine, the latter being dependent on the volition of cocaine self-administration [169]. Furthermore, prior exposure to cocaine “primes” the inducibility of the *FosB* gene resulting in an increase in both mRNA and protein expression in the NAcc following subsequent reexposure after an extended period of withdrawal [171]. Indeed it is changes to the expression of ΔFosB that have been associated with addiction the most, especially as ΔFosB is estimated to mediate 25% of the changes to gene expression in the NAcc following chronic cocaine exposure specifically in the MSNs of the direct pathway (see [168, 172]). Furthermore ΔFosB accumulates in reward-associated nuclei in response to drugs of abuse where it is believed to play a role in mediating reward (via induction of the GluA2 subunit of AMPA receptors) [173], acquisition, and motivation [174]. 

Following extinction training Fos may play a greater role in addiction than ΔFosB as its expression is increased in the basolateral amygdala and prelimbic PFC during this period [175]. This occurs in the absence of changes to GluA2. Furthermore, the induction of Fos transcription factors is heavily influenced by CREB which can be induced by a drug and binds to the FosB promoter. In mice, if CREB and serum response factor are deleted from the NAcc, exposure to cocaine fails to result in the normal induction of ΔFosB and animals become less sensitive to cocaine's rewarding effects [176, 177]. However, deletion of CREB alone has no effect on ΔFosB and actually enhances cocaine rewarding effects. Furthermore, serum response factor appears to be involved in mediating ΔFosB activity following stress-related responses [176] highlighting the complex intracellular regulation of these factors in response to, at least, cocaine.

### 3.3. Epigenetics

While changes to the underlying gene structure may increase an individual's predisposition to addictive behaviours, new insights indicate that specific mechanisms regulate posttranslational modifications of gene expression. This enables the control of heritable changes to phenotypes that are not dependent on changes in the genetic code itself (see [178, 179]). So termed “epigenetics”, these modifications occur via remodelling of chromatin structure incorporating the influence of environmental stimuli. Consequently, enhanced or suppressed transcription of genes to proteins is influenced by the summation of posttranslational modifications to histones or changes to DNA itself independent of the DNA sequence. These modifications limit access of transcription factors and thus result in altered gene expression (see [180] for a review). While every cell contains the same DNA sequence, it is believed that epigenetic mechanisms act to regulate the specific pattern of genes expressed at any given time thus serving to establish and maintain different gene expression programs in specific cell types and imprinting a phenotype on that cell [181]. Epigenetics can act to regulate gene functions in a reversible but stable manner including whether the expression of a gene is turned on or off. This includes priming genes following exposure to a drug, such that subsequent exposures result in increased mRNA or protein expression of the gene product [171]. Epigenetic processes therefore permit long-term regulation of gene function which does not require mutagenic mechanisms. Epigenetic mechanisms are also believed to play a key role in mediating personality traits as indicated from identical twin studies [182] and influence of gene-environment interactions.

Our knowledge regarding the role of epigenetics in addictive behaviours is relatively new (see [178] Table 2 for extensive list of epigenetic changes following exposure to drugs of abuse). A current hypothesis is that exposure to drugs of abuse results in stable epigenetic modifications that alter gene expression and neuroadaptive changes seen during the transition to, and maintenance of, an addicted state. It is also believed that these epigenetic changes act to perpetuate relapse following periods of abstinence [183–185]. Of the possible epigenetic processes known DNA methylation and posttranslational modification of histones are currently the best understood. However, while both these processes have the ability to mediate downstream neuroadaptive changes, they do not appear mutually exclusive.

#### 3.3.1. DNA Methylation

DNA methylation occurs via the addition of a methyl group in a reaction catalysed by a group of enzymes called DNA methyltransferases (DNMTs), of which there are currently 3 members (see [186]). DNMT 3A and 3B are involved in the transfer of methyl groups to naked or unmethylated regions of DNA and are referred to as de novo DNMTs. In contrast, DNMT 1 methylates hemimethylated DNA in the precise manner that mimics the methylation patterns present prior to replication. Thus DMNT 1 is responsible for maintaining methylation during replication and can also repair DNA methylation being referred to as a maintenance DNMT. DNA methylation can occur at any one of the 4 possible bases (i.e., cytosine, adenine, guanine, and thymine); however, methylation of cytosine appears the most stable. In the case of cytosine, methylation occurs primarily (but not exclusively) when it is positioned next to guanine in the DNA sequence (CpG) (see [178]). Methylation in promoter regions, which often contain large numbers of CpG repeats called CpG islands (up to 3,000 base pairs in length), for example, inhibits the ability of transcription factors to bind to the gene. This also involves recruitment of methyl-binding domain containing proteins such as MeCP2 to form corepressor complexes which leads to chromatin compaction and gene repression (see [178, 179]). 

DNA methylation can occur in a region and drug-specific manner [187] and is believed to play a key role in synaptic plasticity, learning, and memory formation [188]. Acute cocaine exposure in mice upregulates the mRNA expression for DNMT 3A and 3B in the NAcc [189]. For DNMT 3A this occurs within 1.5 hours after treatment, with both DNMT 3A and 3B being upregulated after 24 hours. Repeated exposure for 7 days does not alter DNMT expression but at both time points, however, DNA hypomethylation is present at the FosB promoter. This corresponds to an upregulation of FosB in the NAcc and affects the appearance of behavioural sensitization [189]. Interpretation of epigenetic changes, especially in human studies, should note that age alone may alter DNA methylation levels as methylation of DAT increases with age [190]. There also appears to be the possibility of an overlap between epigenetic modifications such as differential CpG methylation and SNPs, with methylation of SNPs for prodynorphin in the PFC being associated with alcohol dependence in humans [191].

#### 3.3.2. Histone Modification

Histones are alkaline proteins which form the chief components of chromatin. They comprise 6 classes subdivided into 5 families (H1/5, H2A, H2B, H3, H4, and H5) with H1 and H5 considered linker histones (to the DNA) and the remainder known as core histones (two copies each) around which DNA is wrapped. Modifications to N-terminal histone tails or globular domains include acetylation, phosphorylation, ubiquitination, sumoylation, methylation, and polyADP-ribosylation, with acetylation being the most well understood (see [179] for more information regarding histone phosphorylation and methylation). Acetylation weakens or disrupts histone to DNA contacts, causing the chromatin to relax, which increases access of transcription factors to the DNA and is thus a sign of active chromatin [185]. 

Drugs of abuse induce specific modifications to histones, this can be gene and region specific and can be heavily influenced by whether the drug is presented either acutely or chronically. This can further be impacted by the duration of exposure, with subsequent and different modifications being observed during periods of withdrawal [178, 179]. Genomewide studies suggest that acute exposure to cocaine, for example, activates most genes acutely by acetylation at H4 [192]. Chromatin immunoprecipitation assays in rats have shown that in the NAcc this can occur at the cFos promoter within 30 minutes but appears transient, disappearing by 3 hours, and nonresponsive to subsequent exposures which is constant with the time course of induction of cFos expression following cocaine. In contrast to the findings for cFos, chronic exposure to cocaine increases and sustains H3 acetylation at the BDNF and Cdk5 promoter for 1–7 days after the final dose [192], supporting the observation of chronic exposures activating most genes by acetylation at H3 (see [180]). Studies of genomewide chromatin changes in the NAcc in mice have shown that more genes are acetylated at H3 compared to H4 following repeated cocaine administration, with few gene promoters displaying acetylation at both H3 and H4 [193]. This indicates that while both modifications may regulate gene expression following cocaine, they act independently on distinct populations of genes. 

Epigenetic changes may also result in neuroadaptive changes that consequently cause altered behaviour. Repeated exposures to cocaine in mice reduces the levels of H3 lysine 9 dimethylation in the NAcc [194]; the authors suggest that histone methylation may play a critical role in neuroadaptations in the NAcc that mediate cocaine preference. While drug-induced histone modifications appear relatively stable, persisting in the PFC for up to 2 weeks into straight abstinence [180], they do not appear global such that changes to acetylation at one family can also be differentially regulated across genes. Acetylation of H3 (K9–14) for the immediate early gene EGR1 is reduced and maintained for up to 10 days of abstinence following chronic cocaine, while H3 acetylation of neuropeptide Y is transiently increased at day 1 of withdrawal returning to base line by day 10 [113]. In both cases, H3 acetylation mimics mRNA expression for EGR1 and neuropeptide Y, respectively. In the VTA, 7 days of forced abstinence from cocaine increases protein levels for BDNF and exon I transcripts which are associated with increased H3 and CREB-binding protein activity [62]. However, interpreting the role of acetylation in addictive behaviours becomes complicated by the fact that epigenetic changes can precede their downstream results by some time. Chronic self-administration of cocaine in rats increases acetylation of the promoter for BDNF in the NAcc within 24 hrs after the last exposure [192]. However, BDNF protein is not elevated until a week after cocaine withdrawal [65], suggesting that epigenetic changes may play a role in priming genes for subsequent induction. 

Understanding the impact of epigenetic modifications is complicated by the fact that these modifications are potentially reversible and can be mediated by other processes. For example, acetylation is highly regulated by two families of enzymes, namely, histone acetyltransferases (HATs), which promote acetylation by catalysing the addition of acetyl groups, and histone deacetylases (HDACs) which remove acetyl groups. These enzymes integrate intracellular signals to regulate activation or suppression of gene programs (see [195] for review). HDACs are able to modulate cocaine's effects on locomotor activity [192] and, when an HDAC inhibitor is infused into the shell of the NAcc in rats, responding for cocaine increases, while overexpression of HDAC4 in the same region reduces responding [196]. Together this would suggest that HDACs have the ability to influence the motivational aspects of self-administration, at least for cocaine. Exposure to a drug is also sufficient to differentially affect the expression of these regulatory enzymes which may result in either activation (i.e, via DARPP-32 or CREB-binding protein (CBP)) or suppression (i.e., via HDAC or DNMT 3A) of transcription [197]. Mutant mice that have depleted levels of the HAT CBP show less behavioural sensitization to cocaine following 10 days of exposure, which correlates with decreased histone acetylation and FosB expression (though FosB expression was normal prior to cocaine) [198]. Prior drug exposure may also prime the response to another drug of abuse by enhancing the transcription of FosB, through inhibition of HDAC, and enhancing the depression of LTP in the NAcc [199]. There is also a close association between the regulation of gene expression by transcription factors or via epigenetic modification with interplay between the activating or repressing effects of a transcription factor and chromatin-mediating enzymes at certain genes. For example, phosphorylation of CREB recruits CBP. As CBP is a HAT it is able to acetylate histones thus increasing gene expression, while also being a transcriptional coactivator regulating the expression of ΔFosB (see [200]). ΔFosB can also regulate lysine dimethyltransferases, which mediate histone methylation and contribute to dendritic spine plasticity and cocaine preference [194].

While only DNA methylation and histone acetylation are presented in this paper and are considered separate identities, crosstalk exists between these factors making epigenetic modifications highly complex and integrated. Consequently, it is very unlikely that only a single process occurs at any one given time point. Transcriptome profiling in postmortem brain tissues from the amygdala and frontal cortex in 17 alcoholics reported both DNA hypomethylation and histone H3K4 trimethylation [201]. These processes can occur concomitantly at the same gene. Chronic intermittent exposure to ethanol results in DNA methylation [202] and H3K9 acetylation of the 5′ regulatory region of the gene for GluN2B, the latter occurring predominately during withdrawal and coincides with a significant decrease in H3K9 methylation [203]. Furthermore, H3K9 methylation can direct subsequent DNA methylation [204] with decreased histone methylation corresponding to altered gene expression in the PFC (especially those genes coding for cell adhesion molecules and transcription factors) and altered behaviour in adult rats following 12 days of cocaine at ascending concentrations during adolescence [205].

### 3.4. Noncoding RNA

Conventionally DNA produces mRNA transcripts which are then translated into proteins. However it appears that the majority of genomic DNA is transcribed into what is now referred to as noncoding RNA (ncRNA). This allows RNA itself to act as a regulator of subsequent mRNA translation. It is becoming increasingly apparent that ncRNAs play distinct roles in determining many cellular processes by modulating inhibition or activation of gene expression in processes including regulation of cell cycling, differentiation, signal transmission, apoptosis, synaptic plasticity, and response to DNA damage (see Figure  1 in [206]). To date, ncRNAs include microRNA (miRNA), endogenous small interfering RNA (siRNA), short hairpin RNA (shRNA), piwi-interacting RNA, small nucleolar RNA, ribosomal RNA, splice junction-associated promoter-associated short RNA, termini-associated short RNA, and large intergenic ncRNA, among others [206, 207]. These ncRNAs can be classified as either small (<400 nucleotides) or large (>400 nucleotides) and contribute to infrastructure (i.e., ribosomal RNA) or regulatory (i.e., miRNA) mechanisms. They can interact at the level of either DNA, RNA, or proteins themselves affecting transcription, translation and the stability of mRNA, alternative splicing and epigenetic regulation [206, 207]. 

The expression of ncRNAs can be regulated in an activity-dependent manner, and while there is increasing information about the role ncRNAs play in neurodegenerative disorders and disease, the function of many of these ncRNAs is not well defined especially *in vivo*. Indeed their role in addictive behaviour is relatively unexplored though there is growing evidence that they may play significant roles. For example, in rats introduction of shRNAs in the NAcc shell is sufficient to downregulate endogenous CREB expression and reduce cocaine reinforcement [45]. As our ability to utilise ncRNAs to determine the functional roles of pathways is increasing, ncRNAs appear to provide a potential avenue for therapeutic intervention (see Section 3.4.2 below). This is in light of new technology incorporating the use of recombinant adenoassociated virus vectors to introduce ncRNAs into specific neural pathways. These vectors can be injected intravenously to target and inhibit selected ncRNAs thus increasing mRNA levels of their targets and permitting investigation of their function [208]. Their unique feature includes long-term stability having been shown to maintain inhibition of ncRNAs for at least 25 weeks [208]. Furthermore, these vectors overcome the prior limitations requiring repeated administration and route of delivery seen with complimentary chemically modified anti-ncRNA oligonucleotides which have been used in the past.

#### 3.4.1. MicroRNA

MicroRNAs are short endogenous ncRNAs (ranging in size from 19 to 25 nucleotides) that coordinate the fine tuning of posttranscriptional gene expression, first discovered for their role in development [209]. Long primary miRNA transcripts are initially processed to short pre-miRNAs which are transported from the nucleus and cleaved to short nucleotide sequences. Via interactions with the enzyme Dicer, which facilitates the formation of the RNA-induced silencing complex, precursor miRNAs become functionally binding to complementary sequences of 3′-untranslated regions of their target mRNAs. This results in the repression of translation of mRNAs and either inhibits protein synthesis or induces sequence-specific degradation of mRNA at the posttranscriptional level [210–213]. Evidence also suggests that miRNAs, such as miR373 which is involved in the regulation of E-cadherin, may also act to enhance gene expression [214]. There are currently hundreds of known miRNAs and their complexity is amplified by the fact that any one miRNA has multiple targets, and more than one miRNA can regulate the same mRNA [215]. 

MiRNAs have clear functions in development [209] including neurogenesis, with further roles in synaptogenesis and plasticity. This includes the regulation of receptor expression [216] and function [217], as well as regulating LTP and LTD [218], especially in hippocampal-mediated memory and learning [219]. Indeed the induction of either LTP or LTD is sufficient to evoke changes in the expression of numerous hippocampal miRNAs [220]. While the expression of most miRNAs in the hippocampus may be regulated by either LTP or LTD and occurs with a similar expression profile, they display different expression dynamics including the time at which expression occurs [220]. 

Since miRNAs (1) mediate dopaminergic responses [221], (2) alter receptor expression (including glutamate receptors) [222], (3) have the ability to respond rapidly to cellular signals to regulate local mRNA expression, and (4) display subcellular localisation, including dendrites, they are believed to play a key role in the conversion of drug-induced synaptic plasticity to long-term adaptations. Both the expression of dopamine D_1_ receptors [221] and the fine tuning of dopaminergic processes and dopaminergic-mediated behaviours such as locomotion [223] can be regulated by specific miRNAs. Furthermore, drugs of abuse can alter gene expression via miRNA-mediated pathways [224], with miRNAs implicated in mediating the effects of cocaine [225], alcohol (see [226, 227] for extensive review), and nicotine [224]. It has been shown that nicotine specifically alters the expression of up to 25 miRNAs including an increased expression of miR-140 which inhibits, among others, DNMT 1 [224]. Acute alcohol up regulates miR-9, which has been linked to tolerance via its effects on posttranscriptional reorganization in the mRNA for calcium- and voltage-activated potassium (BK) channels [228]. 

However, exposure to a drug can have differential effects on miRNA expression. Chronic cocaine treatment in rats can both up- (miR-181a) and down- (miR-124 and let-7d) regulate the expression of miRNAs in a miRNA- and region-specific manner [225], including in the NAcc, dorsal striatum, and hippocampus. It can also both decrease and increase over 30 miRNAs specifically localised to post-synaptic densities [229]. The three miRNAs mentioned above have the ability to directly affect drug-related behaviours such as attenuating (miR-124 and let-7d) or enhancing (mi-181a) a CPP to cocaine when expressed in the NAcc [230]. Furthermore, expression of miR-124 and let-7d alters BDNF and dopamine receptor expression at the protein level [225], with miRNA-induced alterations in BDNF resulting in altered dendritic spine morphology [231]. The miR-124 also targets the transcription factor RE1-silencing transcription factor (REST) thus repressing gene expression in a double negative feedback loop manner [232]. REST can also suppress BDNF indicating that there is a complex interaction on the inhibition of BDNF that can occur at both the translational (miRNA) and transcriptional (REST) level. 

While miRNAs can alter the expression of neurotrophins, miRNAs can also be induced by neurotrophins. In neonatal rat cortical neurons, the presence of BDNF upregulates the precursor miRNA-132, a target and inhibitor of CREB, which is sufficient to affect neuronal morphogenesis and increase neurite outgrowth [233]. Via a different CREB-mediated mechanism, miR-212 expression in the dorsal striatum alters the stimulatory effects of cocaine [234]. Using lentivirus the authors show that overexpression of striatal miR-212 decreases the motivation of animals to self-administer cocaine, while antisense oligonucleotide inhibition of the same miRNA increases cocaine intake [234], suggesting that deficits in miR-212 signalling may increase an individual's vulnerability to addiction. 

It is also becoming evident that miRNAs can mediate chromatin remodelling through the regulation of factors such as MeCP2, a DNA-binding protein that can compact chromatin structure independent of DNA modification such as methylation [235]. Activation of MeCP2 may not only result in repression of transcription [236] but may also activate transcription through CREB [237]. Chronic ethanol for 10 days can alter the expression of 26 miRNAs in cultured cortical neurons, 20 of these being differentially expressed should ethanol treatment only continue for 5 days followed by 5 days of abstinence, 3 of these having the common target MeCP2, suggesting that cessation of drug influence is an independent factor regulating miRNA expression [238]. Furthermore, MeCP2 and miR-212 can form homeostatic interactions in the dorsal striatum that alter cocaine's effects on BDNF levels [239]. Thus altered expression of miRNAs appears sufficient to drive specific aspects of addictive behaviours via intricate mechanisms which may occur via direct regulation of protein synthesis at the synapse or via interaction with transcription or epigenetic factors at the cellular level. This can result in the activation of CREB, which promotes transcription, or REST, which suppresses transcription, of the same target; both of these factors can also regulate miRNA expression [240]. Therefore it is hypothesised that it is the combined actions of these three elements (miRNA, CREB, and REST) that ultimately regulate gene expression via coordinated feedback mechanisms.

#### 3.4.2. SiRNA

SiRNAs are small (19–23 nucleotides) double stranded ncRNAs that associate with multicomponent nucleases called RNA-induced silencing complexes. This complex guides the siRNA to the complementary mRNA region resulting in sequence-specific gene silencing. Unlike antisense oligonucleotides, which can block the translation of a single copy of mRNA before being degraded, siRNAs can regulate multiple mRNA transcripts, are 100 times more effective than antisense oligonucleotides, and are sustained for a relatively long duration [241]. Consequently, siRNAs (and shRNAs) are beginning to emerge in the field of neuroscience due to their therapeutic potential. This has initially been shown for treatment of cancer where antisense oligodeoxynucleotides mimicking siRNA/shRNA function can be produced to downregulate gene expression (see [242]). With respect to the same application for treating addiction, siRNAs have been used to stably transfect HEK-MOR cells *in vitro* to silence the expression of CREB and Ets-like protein-1, both targets for activated ERK1/2 [243]. In doing so the cellular responses following stimulation of the cells with morphine and following withdrawal were significantly altered. SiRNAs against the GluN2B subunit of NMDA receptors have also been injected into the NAcc and VTA in rats [244]. The authors used this technique to show that downregulation of the GluN2B subunit for up to 14 days is sufficient to abolish reward behaviour (as measured by CPP) following chronic morphine exposure but not behavioural sensitization to repeated exposures, though this occurred only when siRNAs were injected into the NAcc and not the VTA. Indeed, a recent study by Bonoiu and colleagues used nanotechnology to produce a gold labelled nanorod-DARPP-32 siRNA complex in an attempt to target dopaminergic signalling in the brain [245]. While their experiments were conducted *ex vivo*, they were able to show uptake of these antagonist nanoplexes in dopaminergic neurons which resulted in functional gene silencing of dopamine and cAMP-regulated phosphoprotein-32 for up to 1 week after transfection and other key downstream effector molecules of this pathway including ERK and phosphoprotein phosphatase 1. The nanoplexes did not appear to result in cytotoxicity and were shown to transmigrate across an *in vitro* blood brain barrier model [245]. While numerous considerations exist for implementing ncRNAs in RNA interference-based gene therapy including route of delivery, kinetics, and offtarget effects (see [246] for extensive review), they nonetheless provide the potential for site-specific approaches for treating drug addiction. Indeed they have already been used to knock down gene expression for TrkB within the NAcc in rats which is associated with inhibition of a CPP and reinstatement to cocaine [66] and with lentivirus mediated shRNA knock-down experiments indicating that CaMKIIalpha expression in the NAcc (shell) plays a role in the motivation to self-administer cocaine [196].

### 3.5. Optogenetics

To date dissection of the microcircuitry controlling aspects of addictive behaviours has, in part, relied heavily on the use of lesion or electrical stimulation experiments, pharmacology studies employing exogenous application of antagonists/agonists administered either peripherally or localised to a discrete nucleus, or the generation of knock-out or knock-in animals, some requiring further genetic manipulation and temporal activation via secondary drug application or electrical stimulation (see [247, 248]). Each of these approaches provides information relative to the mechanisms mediating addiction but contain unavoidable drawbacks. Antagonist experiments are dose and time dependent, require drugs to be degraded before normal activity can be returned, may produce secondary effects at other targets, and do not allow the generation of defined patterns of spike wave activity. Knock-out/in animals may result in secondary compensatory changes as a result of altered gene expression throughout development. While conditional knockout/ins provide some improvement to this issue, the effects of altered gene expression cannot be independently regulated. Electrical stimulation is complicated by the fact that many cells are deeply embedded in dense heterogeneous brain tissue making selective stimulation difficult based on microelectrode placement and may result in costimulation of fibre networks within the targeted region. The technique often requires numerous electrodes at once which may result in secondary tissue injury.

New advances in technology have seen a growing application for optogenetics in neuroscience research. The concepts on which our current use of optogenetics is based were first described by Boyden and colleges in two papers originating in 2005 [249] and 2006 [250]. The technology was subsequently reviewed in 2006 [251] with the first *in vivo *demonstration of the ability to alter behaviour in rodents via excitation of motor cortical neurons by the same group in 2007 [252]. Hailed as the method of the year by Nature in 2010 [253], optogenetics combines theories from optics, genetics, and bioengineering to study the function of intact neuronal circuits to gain greater insight into neural dynamics and behaviour. Optogenetics also appears to have many advantages as it has high temporal precision being utilised in awake behaving animals. This enables causal connections between specific neuronal populations and behaviour output to be investigated allowing distinction between the function of neuronal microcircuitry. It has been demonstrated in *C. elegans* that this technology can be used to induce the release of specific neurotransmitters at neuromuscular junctions to alter behaviour [254]. Thus via optogenetic stimulation in this model release of acetylcholine induces body contraction, whereas stimulation of GABA release results in body elongation. Optogenetic probes are primarily versions of opsins, seven-transmembrane ion channels that are light sensitive and subsequently translocate ions across plasma membranes upon stimulation. There are 3 main classifications of these probes based on the results following stimulation, those that result in depolarization (i.e., channelrhodopsin-2 (ChR2)), those that result in hyperpolarisation (i.e., halorhodopsin) and those that alter intracellular signalling (see [247]). 

#### 3.5.1. Neuronal Excitation

To date the majority of work has focused on optogenetics using the green algal protein ChR2, a light activated nonspecific cation ion channel. ChR2 can be introduced at the level of the cell body and, once transfected into the membrane of the target cell, will induce cell firing upon stimulation. Alternatively, ChR2 can be introduced to neuronal axons and synaptic terminals to investigate the strength of afferent-dependent synaptic transmission and pathway-specific neurotransmitter release [255]. Targeting is facilitated via fusion of ChR2 with cell or neurotransmitter-specific promoters [254] via the use of either transgenic animals that express the protein of interest under cell-specific neural promoters or via recombinant retroviral vectors that are stereotaxically delivered to the target or, to a lesser degree, using electroporation and anatomical-based gene targeting (see [247]). Light sources that include halogen lamps, LEDS, or lasers are then delivered close to the target primarily through implanted optic fibres (deep tissues) or FEDs if superficial activation is required. ChR2 is then activated via the delivery of millisecond (usually 1–5 ms) pulses of blue light (approx. 450–500 nm) thus resulting in a large inward flux of sodium, potassium, hydrogen, and calcium ions and depolarization of the neurons resting membrane potential. This results in photo stimulation of the target cells resolvable to single action potentials, without directly affecting the activity of neighbouring cells (see [256, 257] for in-depth explanation of methods and methodological considerations). Recovery and thus pore closing occur within milliseconds once the light is removed. 

Optogenetics holds many features over other techniques as ChR2 can be genetically targeted to specific defined neural populations within a single circuit which allows accurate, fast (within milliseconds), sustained, and reproducible (with respect to spike firing) stimulation of a large number of neurons [249, 250]. The magnitude of postsynaptic currents (i.e., excitation) is controlled by the duration of the light pulses [250]. Furthermore, as ChR2 requires continuous illumination of high-intensity light to keep the channel open, development of new “slow” variants of ChR2 with residue mutations is being developed. These variants display slower offkinetics, thus delaying channel closing, and increasing light sensitivity (i.e., they are responsive to yellow as opposed to blue light) [258]. By using the ChR2 variant to prolong depolarization, the authors found that they could manipulate long-lasting behavioural alterations and alter developmental stages in *C. elegans*. Additional modifications that can be activated and sustained by one light intensity and turned off with a differing light intensity are also being investigated [247], though modified ChR channels have not yet been successfully demonstrated *in vivo *in mammals. Double mutants of ChR2 have also been produced, so-called stabilized step-function opsins, these variants have greater stability in their depolarization and increased light sensitivity (see [259] for review). A second channel rhodopsin, VChR1, from the algae *Volvox carteri*, has also been utilised to stimulate neurons. The feature of VChR1 is that it is activated by yellow light (590 nm) thus increasing the potential of stimulation of deeper brain areas and co-expression with ChR2 to permit selective activation of 2 targets within the one region [260]. However, as deactivation of VChR1 is relatively slow (compared to ChR2) and VChR1 is also semisensitive to blue light, further development of VChR1 is needed to optimise its potential [248].

#### 3.5.2. Neuronal Inhibition

Using similar principles to those applied to ChR2, modified variants of the bacterially derived light-sensitive chloride pumps halorhodopsin or archaerhodopsin-3 can be used in inhibition studies to silence neuronal activity (see [37, 247]). Halorhodopsin, for example, once activated by yellow light, results in an inward flux of chloride ions, hyperpolarization, and neuronal inhibition (either knockout of single action potentials or blockade of spiking) [261]. In comparison, archaerhodopsin-3 works via light-driven proton pumps. Rhodopsin has also been used to form G-protein coupled receptor chimeras (termed OptoXRs) [262] that selectively recruit distinct biochemical signalling pathways in response to light. Initially the intracellular loops of rhodopsin were replaced with those of adrenergic receptors *α*_1A_ or *β*_2_. In these OptoXRs, 60 seconds of green light are sufficient to drive downstream signalling and functional expression. Their validity was tested in the NAcc in this study where they exerted opposing effects on spike wave activity with opto-*α*_1A_ adrenergic receptors, receptor expressing networks increasing firing, and opto-*β*_2_ decreasing firing [262]. Furthermore the authors found that stimulation of photo-*α*_1A_ adrenergic receptors in the NAcc in freely moving mice leads to an increase of CPP to the environment paired with optical stimulation, specific to reward-related behaviour and not anxiety or locomotor activity.

### 3.6. Optogenetics and Addiction

While the application of optogenetic technology to the study of addiction shows great promise, relatively little work incorporating this technology has been published to date in this field. Indeed optogenetics has been used to stimulate dopaminergic cell firing [263] and dopamine release [264] in the striatum, including the NAcc, in the rat when neurons in either the VTA [263] or substantia nigra [264] are targeted. In this second study the authors were able to fine tune and, respectively, evoke dopamine release ranging from as small as 50 nM to in excess of 500 nM. The kinetics of this simulation mirrored that of electrically evoked release with little observed effect on blood flow as pH was not altered [264]. The technique has also been used to demonstrate that dopaminergic neurons in the VTA release not only dopamine but also glutamate sufficient to elicit excitatory post-synaptic responses in the NAcc [265], suggesting that the mesocorticolimbic reward system may directly involve both dopaminergic and glutamatergic responses, the latter originally thought to play a more modulatory role over this pathway.

The real benefits of optogenetic technology in addiction become apparent via the ability to mimic the activation of circuitry responses to reward-based tasks. Behavioural modification including induction of a CPP have been demonstrated via the use of optogenetic stimulation of dopamine neuronal firing in the VTA [263]. Phasic activation of these neurons in mice enhances reinforcement in operant tasks to a food reward and following extinction food-seeking behaviours can be reinstated via photo stimulation of dopaminergic neurons in the absence of external cues [266]. Optogenetics has also been used to investigate the causal relationship between dopaminergic cell firing and reward, supporting acquisition and maintenance of instrumental responding to food rewards in genetically modified rat lines [267]. Kim et al., have shown that a single transient activation of VTA neurons in mice, mimicking the nature of responses seen following receipt of a natural reward, for as little as 200 ms following self-initiated nose pokes is sufficient to induce operant reinforcement and potentially drive reinforcement learning [268]. Optogenetics has also been used to dissect these circuits in the context of reward. Lobo et al. showed that activation of D_1_ receptors, that is, the direct pathway, in the NAcc enhances cocaine reward whereas activation of D_2_ receptors in the NAcc suppress cocaine reward. The effects of receptor activation on cocaine reward were shown to be similar to those mediated by selective deletion of the TrkB receptor and, in the case of D_1_, alter downstream markers of BDNF-TrkB signalling (i.e., pERK) [269]. Together these studies increase our understanding of the circuit level control of BDNF, TrkB, and dopamine receptor interactions over cocaine reward. 

In 2010 Witten and colleagues demonstrated the first behavioural loss of function relative to addiction using optogenetics in freely moving mice. In this study they demonstrated that cholinergic interneurons in the NAcc play a role in mediating a CPP to cocaine with cholinergic neurons being responsive to cocaine and being able to influence the firing rate of MSNs in this region [270], while still in its infancy, based on the fact that advances in optogenetic techniques permit either short or sustained cell excitation or inhibition, that is, rapidly reversible in awake behaving animals, it has great potential to increase our understanding of the processes mediating addiction. This occurs via the ability to functionally dissect out cell populations and pathways mediating addictive behaviours and which to target for intervention strategies. This is enhanced by the fact that different optogenetic probes can be stimulated in light of different wavelengths meaning they can be incorporated at the same time in the same pathway (see [247] Figure 4 for summary). This occurs without the secondary effects of the other techniques mentioned above or long-term injury and/or adaptive mechanism occurring in the cell. Thus optogenetics holds great potential for application to systems neuroscience. As reviewed by Tye and Deisseroth, by using combinations of expression and illumination sites investigators can determine the effects of activation of local nuclei and their projection sites, the role of specific cell populations within this nuclei, or temporal separation of mixed populations within the same nucleus, or projections to this nucleus (see [259] Figure 2). Furthermore, optogenetics can be incorporated into other techniques to improve the quality of the information obtained. Studies have already combined optogenetics and electrophysiology techniques in order to verify the effectiveness of transgene expression, such that light responsive cells increase and/or decrease spike wave recordings [271]. These combined techniques can also enhance the ability to identify the subpopulation of neurons being recorded and permit noninvasive manipulation of the activity of cells and/or networks during recording sessions (see [272]). Optogenetics has also been incorporated with functional magnetic resonance imaging (fMRI) (so-called opto-fMRI) using blood oxygenation level-dependent (BOLD) signals to show optogenetic stimulation of CaMKIIalpha-expressing excitatory neurons specifically located in the neocortex or thalamus elicits positive BOLD signals in adult rats [273]. Similar results are seen when layer V neocortical neurons are optically stimulated [274]. In these studies, signals were also recorded in downstream targets highlighting the potential to investigate the circuits recruited by defined local cell stimulation [273], importantly this can be successfully carried out awake animals [275].

## 4. Summary

This paper has highlighted the complex relationship between drug induced neuroadaptations in the brain and addictive behaviours using examples from both human studies and animal models. These neuroadaptations can occur at the cellular, molecular, and (epi)genetic level and are hypothesised to be responsible for the transition to, and persistence of, an addicted state. Following repeated exposure to drugs of abuse synaptic plasticity is believed to play a key role in these adaptive changes, at the level of signal transduction, receptor expression, or synaptic structure. There is growing evidence that genetics and altered gene expression have long-term consequences on addictive behaviours. Changes to gene structure via SNPs may increase an individual's vulnerability to becoming addicted should they be exposed to a drug. There is also a greater understanding of the role of epigenetic mechanisms and ncRNAs in mediating addiction as these factors are responsive to drugs of abuse and can alter the transcription and translation of DNA and RNA, respectively. New techniques such as optogenetics are also evolving which permit investigation into the microcircuitry mediating addictive behaviours. However, addiction is a highly integrated process complicated by differing pharmacological profiles of drugs themselves, individual variation (either biological or genetic), and environmental factors, all of which can influence the mechanisms mediating drug-induced neuroadaptive changes and a person's vulnerability to becoming addicted. Thus we face a major contemporary challenge to elucidate the genetic and molecular identities of factors implicated in the development and maintenance of an addicted state.
